# Supplementary material for: Clinical Characteristics and Risk Factors for Fatality and Severity in Patients with Coronavirus Disease in Korea: A Nationwide Population-Based Retrospective Study Using the Korean Health Insurance Review and Assessment Service (HIRA) Database
Source: Int J Environ Res Public Health. 2020 Nov 18;17(22):8559. doi: 10.3390/ijerph17228559 (PMC7698934; doi:10.3390/ijerph17228559)
Supplement: Supplementary file 1 [file ijerph-17-08559-s001.pdf]

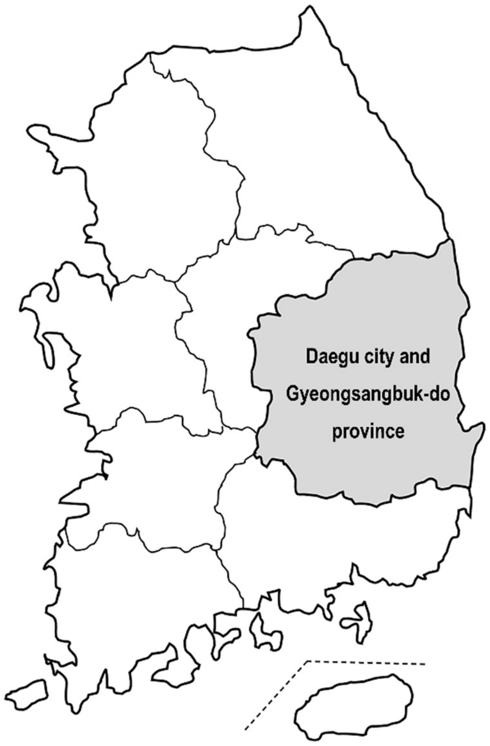

Figure 1. Location of Daegu city and Gyeongsangbuk-do province.

Table S1. Association between each class of antibiotics and death in patients with coronavirus disease-19 infection.

	Univariable Model		Multivariable Model 1 *		Multivariable Model 2 §	
	**OR (95% CI)**	***p* Value**	**OR (95% CI)**	***p* Value**	**OR (95% CI)**	***p*-Value**
Penicillins	25.43 (19.05–33.93)	<0.001	4.36 (2.94–6.49)	<0.001	8.40 (6.04–11.68)	<0.001
Cephlosporins	8.27 (6.21–11.01)	<0.001	1.14 (0.72–1.79)	0.576	2.27 (1.65–3.14)	<0.001
Quinolones	8.19 (6.13–10.94)	<0.001	1.41 (0.94–2.10)	0.098	2.85 (2.06–3.93)	<0.001
Macrolides	4.80 (3.67–6.29)	<0.001	1.14 (0.75–1.73)	0.538	1.43 (1.05–6.69)	0.025
Aminoglycosides	6.99 (3.22–15.18)	<0.001	0.84 (0.27–2.61)	0.764	4.35 (1.63–11.59)	0.003
TMP/SMX	7.39 (3.39–16.11)	<0.001	0.69 (0.21–2.29)	0.541	2.65 (1.05–6.69)	0.039
Tetracyclines	5.70 (2.18–14.90)	<0.001	0.45 (0.11–1.90)	0.276	3.07 (0.91–10.33)	0.070
Others	30.14 (22.06–41.17)	<0.001	3.96 (2.48–6.32)	<0.001	8.76 (6.04–12.71)	<0.001

* Included all variables regarding medications and comorbidities and variables with $p < 0.05$ in the univariable logistic regression analyses. § Included one medication at a time with all variables regarding comorbidities and significant variables in the univariable analyses and one comorbidity at a time with all variables regarding medications and significant variables in the univariable analyses. TMP/SMX: Trimethoprim and sulfamethoxazole.

Table S2. Association between each class of antibiotics and severe disease in patients with coronavirus disease-19 infection.

	Univariable Model		Multivariable Model 1 *		Multivariable Model 2 §	
	**OR (95% CI)**	***p* Value**	**OR (95% CI)**	***p* Value**	**OR (95% CI)**	***p*-Value**
Penicillins	19.69 (16.41–23.64)	<0.001	4.44 (3.49–5.64)	<0.001	10.07 (8.18–12.40)	<0.001
Cephlosporins	10.01 (8.61–11.64)	<0.001	2.02 (1.62–2.54)	<0.001	4.90 (4.14–5.81)	<0.001
Quinolones	8.64 (7.44–10.04)	<0.001	2.05 (1.67–2.52)	<0.001	4.64 (3.93–5.48)	<0.001
Macrolides	5.95 (5.12–6.92)	<0.001	1.39 (1.09–1.77)	0.009	3.00 (2.51–3.58)	<0.001
Aminoglycosides	11.72 (6.39–21.49)	<0.001	1.95 (0.86–4.43)	0.109	12.48 (6.04–25.78)	<0.001
TMP/SMX	27.09 (12.95–56.66)	<0.001	2.16 (0.86–5.39)	0.100	14.46 (6.38–32.75)	<0.001
Tetracyclines	6.61 (3.33–13.12)	<0.001	2.41 (0.65–9.00)	0.191	6.51 (2.67–15.84)	<0.001
Others	39.46 (28.11–55.38)	<0.001	4.39 (2.65–6.74)	<0.001	16.62 (11.38–24.28)	<0.001

* Included all variables regarding medications and comorbidities and variables with $p < 0.05$ in the univariable logistic regression analyses. § Included one medication at a time with all variables regarding comorbidities and significant variables in the univariable analyses and one comorbidity at a time with all variables regarding medications and significant variables in the univariable analyses. TMP/SMX: Trimethoprim and sulfamethoxazole.
